# Patterns of Default Mode Network Deactivation in Obsessive Compulsive Disorder

**DOI:** 10.1038/srep44468

**Published:** 2017-03-13

**Authors:** Óscar F. Gonçalves, José Miguel Soares, Sandra Carvalho, Jorge Leite, Ana Ganho-Ávila, Ana Fernandes-Gonçalves, Fernando Pocinho, Angel Carracedo, Adriana Sampaio

**Affiliations:** 1 Neuropsychophysiology Lab, CIPsi, School of Psychology, University of Minho, Braga, Portugal; 2 Spaulding Neuromodulation Center, Department of Physical Medicine & Rehabilitation, Spaulding Rehabilitation Hospital and Massachusetts General Hospital, Harvard Medical School, Boston, MA, USA; 3 Department of Applied Psychology, Northeastern University, Boston, USA; 4 Life and Health Sciences Research Institute, University of Minho, Braga, Portugal; 5 ICVS-3Bs PT Government Associate Laboratory, Braga/Guimarães, Portugal; 6 CUF Porto Hospital, Department of Psychiatry, Porto, Portugal; 7 Department of Psychiatry, University of Coimbra Hospitals, Coimbra, Portugal; 8 Forensic Genetics Unit, Institute of Legal Medicine, Faculty of Medicine, University of Santiago de Compostela, Galicia, Spain

## Abstract

The objective of the present study was to research the patterns of Default Mode Network (DMN) deactivation in Obsessive Compulsive Disorder (OCD) in the transition between a resting and a non-rest emotional condition. Twenty-seven participants, 15 diagnosed with OCD and 12 healthy controls (HC), underwent a functional neuroimaging paradigm in which DMN brain activation in a resting condition was contrasted with activity during a non-rest condition consisting in the presentation of emotionally pleasant and unpleasant images. Results showed that HC, when compared with OCD, had a significant deactivation in two anterior nodes of the DMN (medial frontal and superior frontal) in the non-rest pleasant stimuli condition. Additional analysis for the whole brain, contrasting the resting condition with all the non-rest conditions grouped together, showed that, compared with OCD, HC had a significantly deactivation of a widespread brain network (superior frontal, insula, middle and superior temporal, putamen, lingual, cuneus, and cerebellum). Concluding, the present study found that OCD patients had difficulties with the deactivation of DMN even when the non-rest condition includes the presentation of emotional provoking stimuli, particularly evident for images with pleasant content.

OCD is a psychiatry disorder characterized by the presence of intrusive unwanted thoughts, images, ideas, and urges involuntarily entering consciousness (obsessions), which the individual tries to neutralize by repetitive behaviors (e.g., checking) or mental actions (e.g., praying) (compulsions). Compulsions produce a temporary relieve from unpleasant thoughts and are negatively reinforced, and thus maintaining the OCD cycle. OCD is probably one of the most disabling psychological disorders with a consistent cross cultural lifetime prevalence of about 2%, and with a typical onset during adolescence^1^.

A variety of neuroanatomic models were proposed to explain the pathogenesis of OCD but they tend to agree on the role of dysfunctional cortico-striatal–thalamus-cortical (CSTC) circuits^2^. CSTC loops project from different regions of the cortex to diverse striatum structures, and from the striatum to the thalamus (via the globus pallidus) and back to the cortex. Different CSTC loops seem to be involved in distinct components of self-regulation (cognitive, emotional and behavioral)^3^. Each CSTC loop has two distinct pathways: a direct excitatory and an indirect inhibitory pathway. In normal conditions, the excitatory pathway is regulated by the inhibitory pathway. In OCD, an overactive excitatory direct pathway is thought to create an imbalance in the CSTC loops^2^.

Despite the consensus on the role of frontal–subcortical loops in OCD, extensive research has been showing that brain regions other than the CSTC loops may contribute to the complexity and diversity of cognitive and emotional deficits in OCD^4^. More recently, several authors have been looking at abnormalities in OCD major brain networks using resting state functional studies^5^. Resting-state functional studies were introduced as a tool for studying distinct brain networks in absence of any specific task demands. By studying the temporal co-activation/connectivity of the different brain regions during resting fMRI acquisition, several “resting state networks” were identified^6^. Among these networks, the Default Mode Network (DMN) has been extensively studied^7^. The DMN, known as a task negative system (i.e., activated during rest condition), is a network connecting medial prefrontal cortex with the posterior cingulate extending to the precuneus, and the medial, lateral and inferior parietal cortex (ITC)^8^. There is now evidence that, besides these regions, there are additional DMN subsystems with core nodes on the dorsal lateral prefrontal cortex and medial temporal lobe^9^. Distinct DMN nodes, each one with specific contributes, sustains internal mentation processes (e.g., self-reflection, mentalizing, auto-biographical memory, imagery). While both anterior and posterior regions of the DMN are associated with self-referential processes, the medial prefrontal cortex node seems to have an important role in social cognitive tasks while the posterior cingulate is associated with autobiographical memory^10^.

DMN shows a higher activation at rest and is usually deactivated when the individual is required to perform a task requiring directing attention to an external stimulus. The efficient deactivation of the DMN was found to be a good predictor of the ability in switching between a self-referential state and a task demanding external focus. In fact, task-induced deactivations of the DMN have been functionally associated with a wide range of goal-directed tasks^11^ and increased cognitive performance^12^,^13^,^14^, supporting the role of this resting state network in maintaining optimal cognitive functioning. Difficulties in deactivating the DMN are evident in several psychiatry conditions^15^,^16^. For example, impaired DMN suppression (medial prefrontal and posterior cingulate cortex) was found in first-episode schizophrenia patients with cognitive impairments during a working memory task^17^. While in schizophrenia, difficulties with the deactivation of the DMN may be associated with excessive demands to a cognitive impaired system, in other disorders difficulties in DMN suppression seem to be related to altered emotional processing (e.g., negative rumination)^18^. For example, in individuals with remitted depression, rumination style was found to be associated with decreased DMN suppression (i.e., anterior-medial prefrontal cortex)^19^.

OCD patients often report problems in disengaging attention from ruminative thoughts or mental images^20^. Contrasting with other anxiety disorders, OCD symptoms are triggered mostly by internal intrusive thoughts or images rather than external stimuli^21^. Despite not having an increased sensitivity to threatening environmental triggers, OCD patients tend to maintain the mental image of those triggers for longer periods, experiencing difficulties in disengaging from this impactful mental representations^22^. Therefore it makes sense to hypothesize that OCD patients may experience difficulties in deactivating the DMN even when the rest condition is interrupted by emotional stimuli intended to trigger obsessive ruminations.

Several studies have been reporting alterations in brain connectivity within the DMN^23^ and between DMN and other resting state networks^24^. For example, Jang *et al*.^25^ reported decreased functional connectivity in OCD between several regions of the DMN (e.g., right anterior cingulate cortex, middle frontal gyrus, putamen) and the posterior cingulate. Consistent with this, Cheng *et al*.^26^ reported a significant negative correlation between symptom severity and functional connectivity from the posterior cingulate. These alterations in connectivity are present not only in OCD patients but also in unaffected immediate siblings^27^. Additionally, failures to deactivate anterior nodes of the DMN (ventral medial prefrontal cortex) in the course of an error-eliciting interference task both were observed in adult^28^ and pediatric OCD populations^29^.

However, the patterns of deactivation in the DMN in OCD in the transition between a resting condition and a non-rest emotional condition have not been consistently studied. The objective of this research is to study the patterns of deactivation in different nodes of the DMN in the transition between a resting and a non-rest emotional task condition consisting on the presentation of images intended to induce harm (unpleasant images) or guilt intrusions (pleasant stimuli).

## Methods

### Participants

Fifteen patients with OCD and twelve healthy controls participated in this study. The following instruments were used in the assessment of all participants: Edinburgh Handedness Inventory (Oldfield, 1971), Structured Clinical Interview for Diagnostic and Statistical Manual of Mental Disorders (SCID-I)^30^, Yale-Brown Obsessive Compulsive Scale” (YBOCS)^31^, and Beck Depression Inventory (BDI)^32^. No comorbid conditions were identified in OCD participants and all of the patients were currently on stable pharmacological treatment. The healthy control group had no history of drug abuse, psychopharmacological medication, and psychiatric or neurological disorders. As expected, the groups were significantly different in terms of OCD symptoms [YBOCS, *t*(15.16) = 13.02, d = 4.77] and depression ratings [BDI, *t*(20.97) = 3.16, d = 1.18]. Demographic and assessment data are presented in Table 1.

Prior to the study, all participants provided written informed consent. The study was carried out in accordance with the declaration of Helsinki and was approved by the Center for Research of Psychology Review Board.

### Stimuli

A total of fifty pictures were selected from the database of International Affective Picture System (IAPS)^33^. The following 20 unpleasant pictures, of different arousal levels, were selected: 3010, 3060, 3064, 3068, 3069, 3100, 3102, 3150, 3168, 3266, 2100, 2110, 2271, 2278, 2312, 2399, 2455,2 490, 2491, 9331 [valence: 2.63 (1.04); arousal: 5.39 (1.38)]. Additionally, 20 pleasant pictures of different arousal levels were chosen: 4643, 4647, 4652, 4656, 4659, 4660, 4670, 4680, 4687, 4800, 2050, 2091, 2154, 2165, 2304, 2310, 2311, 2332, 2360, 2540 [valence: 7.22 (0.56); arousal: 5.37 (1.22)]. Finally, 10 IAPS images were used as neutral stimuli: 7025, 7042, 7052, 7053, 7059, 7090, 7100, 7150, 7175, 7235 [valence: 5.06 (0.29); arousal: 2.81 (0.56)]. Pictures were matched in terms of brightness, contrast, mean spatial frequency and 90% quality JPEG.

The stimuli were presented in an event-related fMRI design (spaced mixed trial), consisting of a 10 second epoch resting condition followed by a 20 second epoch with the presentation of an emotional picture (unpleasant, pleasant or neutral). Stimuli were presented in two separate runs (25 stimuli for each run). Each run lasted for 12.7 min (total paradimg time of 25.4 min). All the presentations were randomized across participants, and within each run. Participants were instructed that different pictures were going to be presented on the screen and that they should be comfortably focusing on the pictures without any additional attention or motor task requirement.

### Image data acquisition

The scanning procedure was carried out in a clinical approved Siemens Magnetom TrioTim 3 T (Siemens Medical Solutions, Erlangen, Germany) using the Siemens 12-channel receive-only head coil. Visual stimuli were presented using an LCD projector Silent Vision Model SV-6011 (Avotec, Inc.) and all the fMRI paradigm was programed in E-Prime 2.0^®^ (Psychology Software Tools Inc.) and synchronized with the MRI scanner pulses.

For structural analysis and registration to standard space, a T1 high-resolution anatomical sequence, a 3D MPRAGE (magnetization prepared rapid gradient echo) was performed with the following scan parameters: repetition time (TR) = 2.3 s, echo time (TE) = 2.98 ms, 160 sagittal slices with no gap, Field-of-View (FoV) = 256 mm, flip angle (FA) = 9°, in-plane resolution = 1 × 1 mm^2^ and slice thickness = 1mm. The scanning parameters for fMRI acquisition were the following: TR = 3 s, TE = 39 ms, FA = 90°, in-plane resolution and slice thickness 3 mm, 41 ascending interleaved axial slices with no gap and FoV = 256 mm. Each run consisted of 254 volumes, and total paradigm scanning time was 25.4 min (2 runs).

### Data Preprocessing and Analysis

Before any data processing and analysis, structural and functional acquisitions were visually inspected for critical head motion and brain lesions. The fMRI data preprocessing was performed using Statistical Parametric Mapping version 8 (SPM8^®^) (http://www.fil.ion.ucl.ac.uk/spm) running under Matlab^®^ R2014b. Images were corrected for slice-timing, motion with realignment processing (images were realigned to the mean image with a six-parameter rigid-body spatial transformation and estimation was performed at 0.9 quality, 4 mm separation, 5 mm full-width at half-maximum (FWHM) smoothing kernel and using 2nd degree B-Spline interpolation), spatially transformed to the standard MNI (Montreal Neurologic Institute) coordinates through an indirect procedure that included: rigid-body registration of the mean functional image to structural scan; affine registration of the structural scan to the MNI T1 template; nonlinear registration of the structural scan to the MNI T1 template using the affine transformation previously estimated as the initial alignment; nonlinear transformation of the functional acquisition to MNI standard space trough the sequential application of the rigid body transformation and the nonlinear warp followed by resampling to 2 mm isotropic voxel size, smoothed to decrease spatial noise with a 8-mm full-width half-maximum Gaussian kernel and high pass temporal filtered (filter width of 128 s).

### Statistical Analyses

**Figure 1:**
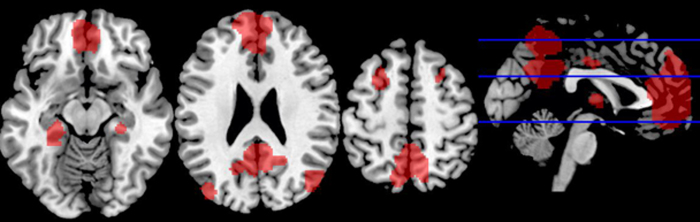
DMN template mask.

In the first level of statistical analysis the resting periods of each participant were convolved with the non-rest periods through a standard deactivation analysis (i.e., a reverse subtraction, ‘resting periods’ minus ‘non-rest periods’, by emotional condition) estimated with the general linear model. The voxel-wise statistical parametric maps, resulted from the contrast described previously, were constrained with a DMN template mask^34^, in order to select only the brain areas within the DMN (see Fig. 1). This DMN mask was provided by GIFT software, and includes most of the brain areas commonly associated with the DMN in the literature. Additionally, an exploratory analysis, contrasting the rest condition with all the non-rest conditions (grouping the three different types of stimuli) was carried out for the whole brain without the DMN mask constrains. Then, the individual statistical functional maps for both groups (OCD vs HC) were entered into a second-level random-effects group analysis using two-sample *t* test analysis, generating a statistical map of the magnitude of group differences (i.e., resting period vs. each emotional task condition). A one sample *t*-test revealed that both groups significantly deactivated DMN and several other regions outside the DMN mask (for the whole brain deactivation analysis). The results were corrected for multiple comparisons p < 0.05 using the Monte Carlo correction. The correction was determined over 1000 Monte Carlo simulations using AlphaSim program distributed with REST software tool (http://restingfmri.sourceforge.net/) with the following input parameters: individual voxel probability threshold = 0.01, cluster connection radius = 3 mm, Gaussian filter width (FWHM) = 8 mm and mask set to the DMN template mask^35^ and the global group deactivation pattern for the whole brain analysis. After magnitude estimation, we decided to include only results with an effect size above 1.1 (Cohen’s d).

## Results

### Patterns of DMN deactivation between OCD and HC

**Figure 2:**
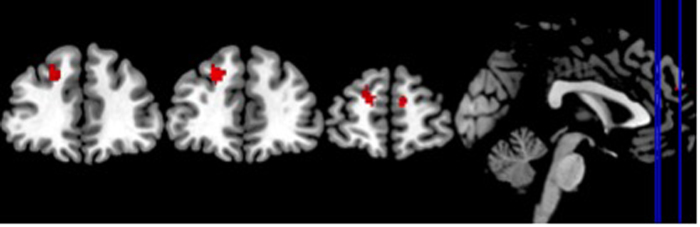
Regions of the DMN significantly deactivated in the HC compared to OCD (Resting state - Rest task-Pleasant Stimuli Condition).

No differences between OCD and HC were observed for the deactivation of nodes of the DMN for all the non-rest conditions (grouped together) as well as the neutral and unpleasant stimuli conditions. However, HC, as compared to OCD subjects, showed an increased deactivation in two anterior regions of the DMN for the pleasant task condition: the right medial superior frontal and the left superior frontal gyri (see Table 2 and Fig. 2).

### Patterns of Whole Brain Resting State Deactivation between OCD and HC

**Figure 3:**
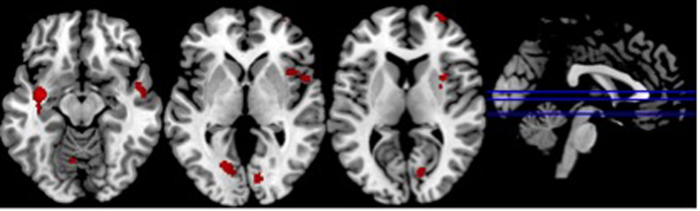
Whole brain regions significantly deactivated in the HC compared to OCD (Resting condition – Non-rest condition).

In order to explore the whole brain unmasked deactivations we compared the rest condition with the non-rest conditions (grouping neutral, pleasant and unpleasant stimuli). Significant increased deactivations were found for the HC, when compared with OCD, in the following regions: right superior frontal gyrus, bilateral insula, left middle temporal gyrus, right superior temporal gyrus, right putamen, bilateral lingual gyrus, right cuneus and left anterior cerebellum (see Table 3 and Fig. 3).

## Discussion

The objective of the present experiment was to research the differences in OCD and HC in terms of patterns of DMN deactivation in the transition between a resting condition and a non-rest emotional condition consisting on the presentation of unpleasant images, pleasant stimuli, or emotional neutral images. Overall the results show that OCD and HC participants did not differ significantly in deactivations on any of the DMN nodes between the rest condition and the non-rest conditions for the all stimuli grouped together as well as for the neutral and unpleasant images. However, when the transition between the resting condition and pleasant emotional condition was considered, HC compared with OCD, showed a significant increased deactivation in two anterior nodes of the DMN: medial frontal gyrus and superior frontal gyrus.

As expected OCD patients experienced generalized difficulties with the deactivation of all DMN nodes in the transition from a resting state to a non-rest condition even when this condition includes stimuli that in previous studies were found to be important emotional triggers for OCD. For example, a previous functional neuroimaging study using identical stimuli, found that OCD patients, when compared with HC, have an increased widespread activation in multiple brain regions to images of negative emotional valence^36^. The present results suggest that these activations may be, at least partially, due to an increased activation already present during the resting state acquisition, probably due to overactive internal ruminations. In fact, previous research with depressive patients, showed that increased connectivity in the DMN was associated with measures of rumination in the rest condition^37^. Additionally, decreased DMN suppression was also related with rumination style in remitted depression^19^.

Impaired DMN deactivation in OCD has been observed in previous studies using emotional-laden tasks. For example, Stern *et al*.^28^ showed that OCD patients had a decreased DMN suppression (ventromedial prefrontal cortex) in an emotion-valuation error-eliciting interference task (monetary contingencies of mistakes). There is now evidence that OCD difficulties with DMN suppression may be due to altered connectivity between the DMN and the Fronto-Parietal Network (FPN)^38^. The capacity for disengaging from ruminative thoughts or internal images may require an efficient connectivity between task negative (i.e, DMN) and task positive networks (i.e., FPN).

An alternative explanation for the lack of deactivation of the DMN is the fact that the active condition did not require any specific attention or motor task. This may have allowed participants to remain in a passive mode, explaining why, also for the HC group, there weren’t any non-rest deactivation other than those for the pleasant emotional condition.

However, in HC the presentation of pleasant stimuli was effective enough for deactivating two anterior nodes of the DMN, the medial frontal and the superior frontal gyri. The medial frontal nodes of the DMN have been associated with self-referential as well as social cognitive processes (e.g., theory of mind)^14^. The present results suggest that in HC, but not in OCD, external demands resultant from the presentation of erotic stimuli were more effective in deactivating internal mentation (self and other) than autobiographical memory processes (posterior nodes of the DMN). The lack of DMN deactivation in OCD for this pleasant condition is consistent with previous findings showing that OCD patients tend to block the processing of erotic stimuli, right from the initial stages of visual processing^22^.

Results of unmasked whole brain deactivation for the non-rest condition (grouping all the stimuli) showed significant deactivations, but again only for the HC group, in several regions widespread across the brain (superior frontal gyrus, the insula, middle and superior temporal gyri, putamen, lingual gyrus, cuneus, and cerebellum). These results are consistent with recent evidence showing that regions outside the DMN may also experience deactivation in response to specific task demands. For example, attention specific deactivations were found in the left ventrolateral and medial prefrontal cortex and the left lateral temporal cortex, while deactivations in right medial-parietal, lateral temporo-parietal, and the medial prefrontal cortex were found in working memory tasks^12^. There is also evidence that an increase in task demand may be responsible for specific deactivation in the insular cortex^11^. Even though no specific task was required in our active condition, one can speculate that the deactivation of bilateral insula may be associated with increased external demand required by presentation of pleasant stimuli leading, in our HC participants, to a reduction of interoceptive self-awareness.

Concluding, the present study found that OCD patients had difficulties in the deactivation of DMN even when the non-rest condition includes the presentation of emotional provoking stimuli, particularly evident in the case of images with pleasant content. The present results should, nevertheless, be contextualized in terms of several methodological limitations. First, no specific motor or attention task was required for the non-task condition. This may have limited the efficacy of the experimental paradigms in triggering a shift from an internal to an external mode. Second, by relying exclusive on a standardized emotional database (i.e. IAPS) the experiment did not control for potential idiosyncratic reactions to emotional images (as well as the impact of images in OCD relevant emotions such as guilt or harm). Additionally, we cannot conclude about the specificity of the present results for OCD. As discussed above, ruminative thoughts were positively correlated with DMN connectivity in other psychiatry disorders. It may be possible that difficulties in deactivating DMN may be transversal to several psychiatry disorders inside or outside the OCD spectrum. Future studies, should attempt to replicate these findings substituting a passive emotional task by an active attention/motor emotional task in a larger sample of patients with ruminative symptoms inside and outside the OCD spectrum.

## Additional Information

**How to cite this article**: Gonçalves, Ó. F. *et al*. Patterns of Default Mode Network Deactivation in Obsessive Compulsive Disorder. *Sci. Rep.* **7**, 44468; doi: 10.1038/srep44468 (2017).

**Publisher's note:** Springer Nature remains neutral with regard to jurisdictional claims in published maps and institutional affiliations.

## Figures and Tables

**Table 1:** Demographic information by group (OCD = Obsessive-Compulsive Disorder; HC = Healthy Controls; EHI = Edinburgh Handedness Inventory, YBOCS = Yale–Brown Obsessive Compulsive Scale, BDI = Beck Depression Inventory).

	OCD	HC
N	15	12
%Female	26.7	33.3
Age	31.67(11.44)	30.92(8.98)
EHI	93.53(7.86)	90.0(14.77)
YBOCS	23.80(6.74)	0.67(1.23)
BDI	9.47(6.63)	3.33(3.17)

**Table 2:** DMN Deactivation (Rest task-Pleasant Stimuli Condition; L-Left hemisphere; R – Right hemisphere; HC – Healthy controls; OCD – Obsessive compulsive disorder).

HC > OCD				
*Region*	*Side*	*Z Score*	*k (Cluster Size)*	*MNI Coordinates (x, y, z)*
Medial Frontal Gyrus	R	3.55	81	10 54 24
Superior Frontal Gyrus	L	3.44	100	−18 44 36

**Table 3:** Whole Brain Deactivations (Rest condition - Non-rest condition; L-Left hemisphere; R – Right hemisphere; HC – Healthy controls; OCD – Obsessive compulsive disorder).

HC > OCD				
*Region*	*Side*	*Z Score*	*k (Cluster Size)*	*MNI Coordinates (x, y, z)*
Insula	L	4.09	110	−38, −4, −12
Middle Temporal Gyrus	L	3.24		−42, −12, −18
Lingual Gyrus	L	3.91	269	−14, −76, −6
Anterior Cerebellum	L	3.27		−8, −66, −10
Insula	R	3.47	269	46, 14, −4
Superior Temporal Gyrus	R	3.25		50, 2, −10
Putamen	R	3.24		32, 8, 4
Superior Frontal Gyrus	R	3.44	98	28, 66, 8
Lingual Gyrus	R	3.25	143	12, −80, −4
Cuneus	R	2.84		14, −74, 8
